# FYU-Net: A Cascading Segmentation Network for Kidney Tumor Medical Imaging

**DOI:** 10.1155/2022/4792532

**Published:** 2022-10-18

**Authors:** Houwei Feng, Xupeng Kou, Zhan Tang, Lin Li

**Affiliations:** College of Information and Electrical Engineering, The China Agricultural University, Beijing 100083, China

## Abstract

Automated segmentation of renal tumors is essential for the diagnostic evaluation of kidney cancer. However, renal tumor volume is generally small compared with the volume of the kidney and is irregularly distributed; moreover, the location and shape of renal tumors are highly variable, making the segmentation task extremely challenging. To solve the aforementioned problems, a cascaded segmentation model (FYU-Net) for computed tomography (CT) images is proposed in this paper to achieve automatic kidney tumor segmentation. The proposed model involves two main steps. In the first step, a fast scan of the kidney CT data is performed using a localization network to find slices containing tumors, and coarse segmentation is performed simultaneously. In the second step, a segmentation framework embedded with the feature pyramid network module is employed to finely segment kidney tumors. By building a feature pyramid structure, targets of different sizes are distributed to be detected on different feature layers to extract richer feature information. In addition, the top-down structure allows the information of the higher-level feature maps to be transferred to the lower-level feature maps, enhancing the semantic information of the lower-level feature maps. Comparative experiments were conducted on the Kidney PArsing Challenge 2022 public dataset; the average Jaccard coefficient and average Dice coefficient of tumor structure segmentation were more than 70.73% and more than 82.85%, respectively. The results demonstrate the effectiveness of the proposed model for kidney tumor segmentation.

## 1. Introduction

**Figure 1:**
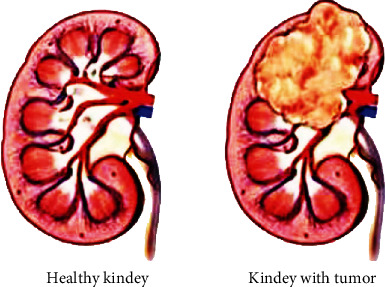
Schematic diagram of kidney tumor.

Image segmentation is used in digital image processing and computer vision to segment an image into multiple parts or regions based on the pixel features in the image [1]. Converting an image into a collection of pixel regions represented by a mask involves separating foregrounds from backgrounds or clustering pixel regions based on similarities in color and shape. Image segmentation is employed in medical imaging for detecting and labeling regions of an image that represent tumors in a patient's brain and other organs. Kidney tumors can be benign or malignant. Benign tumors include cysts, whereas malignant tumors mainly include renal cancer and pelvic cancer [2]. Regular examination, timely detection, and proper treatment are vital to overcome kidney tumors. Kidney cancer can be cured by surgically removing some or all of the organs eroded by cancer [3]. Computed tomography (CT) and magnetic resonance imaging are the most important medical imaging modalities by which surgeons can diagnose and locate kidney tumors [4]. The accurate localization of the kidney tumor is vital in surgery for kidney cancer treatment. Therefore, the precise segmentation of the kidney tumor area plays a crucial role. When successful, it can help the surgeon to locate the kidney tumor area more accurately, thus improving the success rate of the surgery [5]. The structure of the kidney tumor is shown in Figure 1.

In recent years, several deep learning- (DL-) based segmentation models with good performance have been developed [6]; however, organ tumor region segmentation in medical imaging remains challenging, mainly because compared with organs such as the brain or the heart, the segmentation of tumors with large differences in the shape and texture among individuals is more difficult. On the one hand, expert judgment is essentially a decisive consideration, and even with the rapid development of DL and artificial intelligence, medical diagnosis results still depend on the subjective judgment of doctors. However, manual segmentation by doctors is time-consuming and can cause mental fatigue in doctors, which can easily lead to operational errors. On the other hand, unlike organ-based segmentation, the shape and texture of different tumors vary greatly, and finding the commonality between them by direct matching is difficult. Therefore, distinguishing tumors by using organ class-oriented segmentation methods is challenging. Although DL methods have shown great potential in dealing with natural image semantic segmentation problems, some problems are encountered in the field of medical image segmentation, such as poor contrast between tissues and organs and muscles and fats, weak information of segmentation target tissue boundaries, and insufficient feature extraction due to rich information of medical image dimensions [7].

Therefore, in this paper, a cascaded segmentation network for medical images, called the FYU-Net model, is proposed. The proposed model incorporates the idea of cascade segmentation refined from DL computer vision. Comparative experiments were conducted on public datasets, and the results demonstrated the effectiveness of the proposed model for kidney tumor segmentation. The main contributions of this paper are as follows:

- A kidney tumor cascade segmentation model (FYU-Net) is proposed to achieve accurate segmentation of kidney tumor regions. A localization network model is used to identify the sections with tumors from kidney CT data to realize rapid localization of kidney tumor regions and coarse segmentation of tumor regions to be used as the input of the segmentation network
- In the kidney tumor segmentation model, the feature pyramid network (FPN) module is introduced to fuse low-resolution feature maps with strong semantic information and high-resolution feature maps with weak semantic information but rich spatial information with less computational effort. Finally, a classical coder–decoder structure is used to construct a rich pair of medical image information for the accurate segmentation of kidney tumors

## 2. Related Works

Image segmentation is considered the most important medical imaging process wherein regions of interest are extracted using a semiautomatic or automatic process [8]. The image is divided into multiple regions based on a specified description, such as segmenting body organs and tissues for boundary detection, tumor detection, segmentation, and mass detection. Medical image segmentation involves the identification of areas of organs or lesions from medical images to provide the medical community with key information about the shape and volume of body tissues and organs and is one of the most challenging tasks in medical image analysis [9]. In recent years, semantic segmentation has been extensively studied in the field of biomedical imaging.

The Attention U-Net network proposed by Oktay et al. is a novel attention mechanism model for medical imaging segmentation [10]. The model uses an attention mechanism to suppress irrelevant regions in the input image and highlights salient features that are useful for a specific task and can replace hard attention in classification tasks and localization modules in organ localization tasks. Jin et al. proposed RA-UNet, a three-dimensional (3D) hybrid segmentation model based on residual attention perception [11]. The model accurately extracts the volume of interest of the liver and segments the liver tumor from it. The model has the basic structure of 3D U-Net that enables combining the underlying feature mapping with the higher-level feature mapping to extract contextual information and is the first use of residual attention mechanism for medical image processing. Gu et al. proposed CE-Net, a context encoder network model that can capture high-level information and retain spatial information in two-dimensional medical image segmentation [12]. The model consists of three main modules: feature encoding module, context extraction module, and feature decoding module. The U^2^-Net model proposed by Qin et al. is a two-level nested U-shaped structure that captures contextual information from different scales and uses pooling operations to increase the depth of the overall architecture without significantly increasing the computational overhead [13]. The DeepLab V3 model proposed by Chen et al. reexamines the application of Atrous convolution in semantic segmentation and fuses image-level features into ASPP modules, which can obtain contextual information by using the framework of cascade modules and spatial pyramids [14]. DeepLab V3+ is an improved version of DeepLab V3 containing an additional decoder module to correct the segmentation results and further explores the combination of Xception and deep separable convolution with ASPP and decoder modules [15]. He et al. proposed an adaptive pyramid context model (APCNet) that is multiscalable, adaptive, and has global-guided local affinity (GLA) and solves the problem of how to weight the context vector and the original feature map [16]. Zhao et al. developed a pyramid pooling module and a pyramid scene parsing network (PSPNet) to achieve global contextual information aggregation based on the contextual aggregation capability of different regions, providing a superior framework for pixel-level prediction [17].

However, the aforementioned models have certain limitations in tumor segmentation. Due to the need to use the whole CT data as the input of the model and the relatively small size of kidney tumors, the segmentation results may be interfered with by the other regions of the kidney. In addition, these methods do not deeply consider the unique features of medical images during feature extraction, which affects the segmentation accuracy. In this study, we developed a cascaded segmentation network (FYU-Net) that consists of two interrelated steps, namely, localization and accurate segmentation of the kidney tumor region. First, we used the target localization network [18] to extract the slices containing tumor regions from the kidney medical image data and completed the coarse segmentation operation of the tumor in the slices. In the postsegmentation network, we added the FPN [19] module based on the U-Net [20] and fully considered the special features of medical images when extracting the kidney CT data, thereby improving the segmentation accuracy.

## 3. Methods

**Figure 2:**
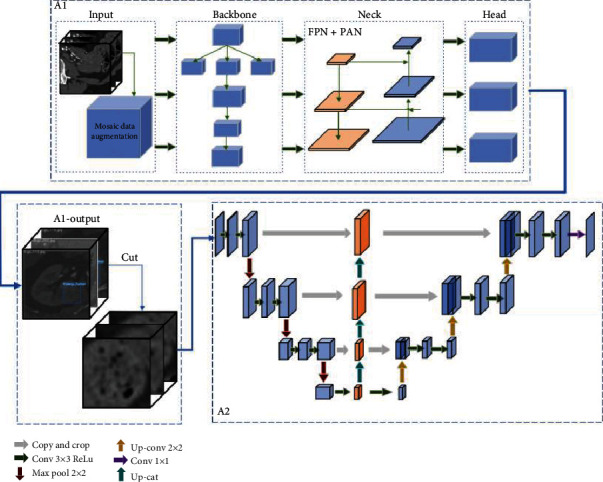
FYU-Net network structure.

The framework of the proposed cascaded segmentation method is shown in Figure 2. Kidney CT data slices are rich in information, whereas the target tumor region is relatively small in size. For this reason, in this study, the kidney tumor region was first extracted and implemented as coarse segmentation, and then, the data obtained after coarse segmentation was used as the input segmentation network for fine segmentation. The first stage of the cascade framework, denoted as A1, was implemented using the YOLO-V5 model to find the slices containing tumors from the total kidney CT data to simultaneously achieve fast, automatic localization of kidney tumors and coarse segmentation of tumors in these slices. In the second stage of the cascade network (denoted as A2), a segmentation network model embedded in the FPN module was used to precisely segment the slices detected from A1 and the corresponding tumor regions based on them.

### 3.1. Location Network

**Figure 3:**
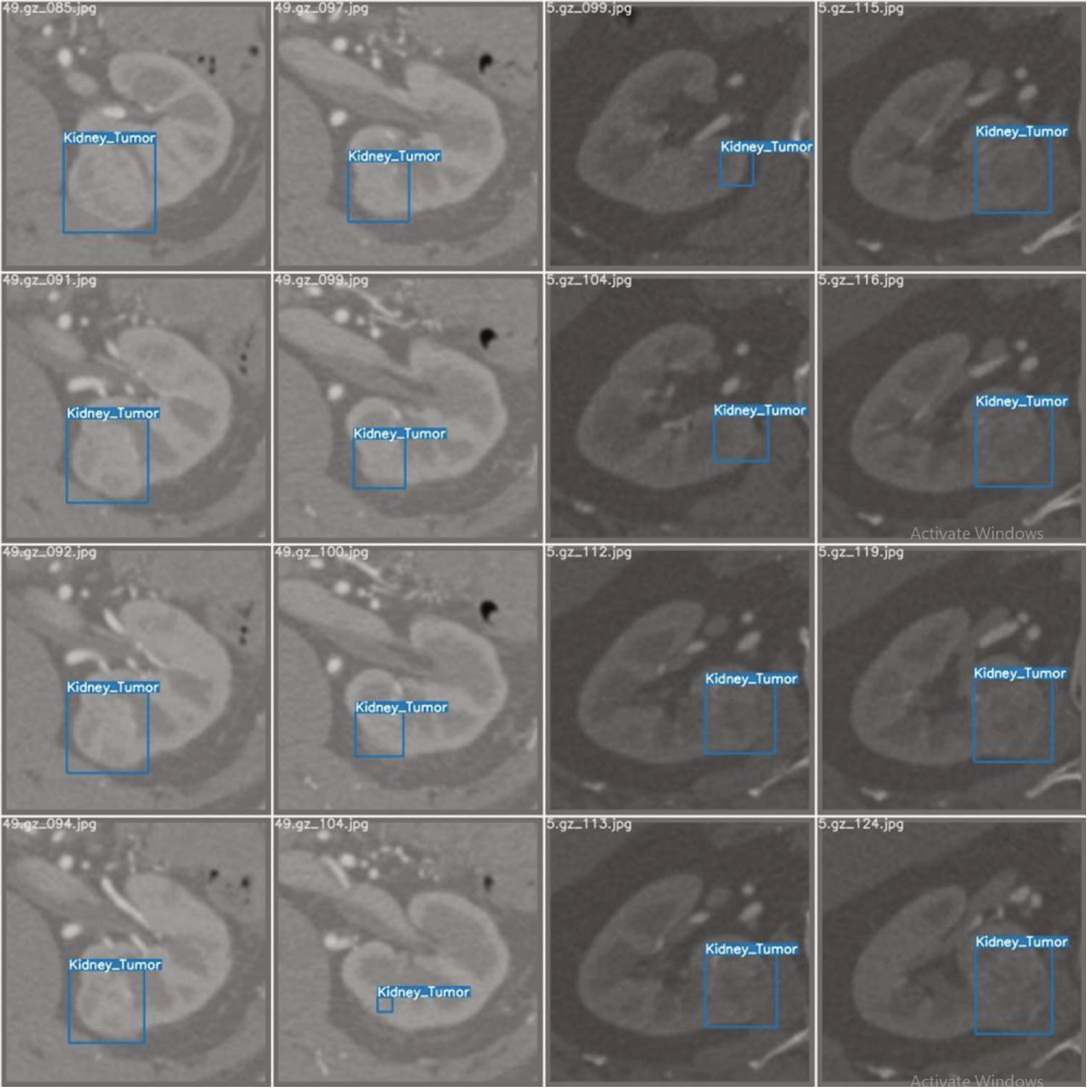
A1 positioning network results.

Due to the rich dimensional information of the original kidney CT data, the slices containing tumor information only account for a small portion of the total number of kidney slices. Tumor regions in the kidney CT data are automatically located in the first stage of the cascade framework (denoted as A1), which provides input data for the coarse segmentation of kidney tumors in the second stage of the cascade framework (denoted as A2). This task can be considered a binary classification problem. Mainstream localization networks include YOLO-V5, YOLO-V4 [21], SSD [22], and Faster R-CNN [23] networks. Among them, YOLO-V5 has the best performance. Therefore, in this study, we used the YOLO-V5 network model for tumor localization. The CT images containing the tumor region detected by the A1 network are shown in Figure 3. After the automatic localization of the kidney tumor region, the images are cropped by expanding ten pixel points outward, according to the detection frame, to achieve a coarse segmentation of the tumor region as the input data of A2.

**Figure 4:**
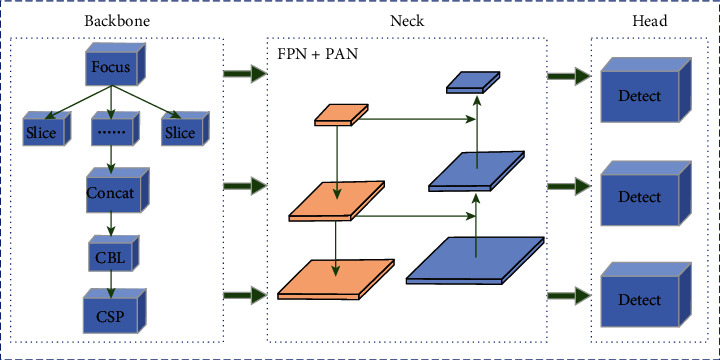
Detailed structure of A1 positioning network.

In the initial stage of A1 localization network training, the mosaic data enhancement operation is used to enhance the training speed and accuracy of the model. Its backbone uses the focus module with a CSP structure to reduce the repetition of gradient information when using the BP algorithm, thereby reducing the computation complexity and number of parameters and improving the learning ability of the network. The focus module is used to slice the image before it enters the backbone, and the CSP structure divides the original input into two branches, CSP1_X and CSP2_X. CSP2_X replaces the Resunit with 2× X CBLs relative to CSP1_X. The CSP structure helps reduce the model size while effectively alleviating the gradient disappearance problem. The neck's network structure contains FPN + PAN for fusing features of different dimensions. FPN uses top-down lateral connections to construct a high-level semantic feature map and a feature pyramid structure; however, because the target information at the bottom layer becomes fuzzy after a multilayer network, PAN adds bottom-up routes to compensate and enhance the localization information. The head structure outputs the target detection results and consists of three detectors. The network architecture of A1 is shown in Figure 4.

### 3.2. Segmentation Network

**Figure 5:**
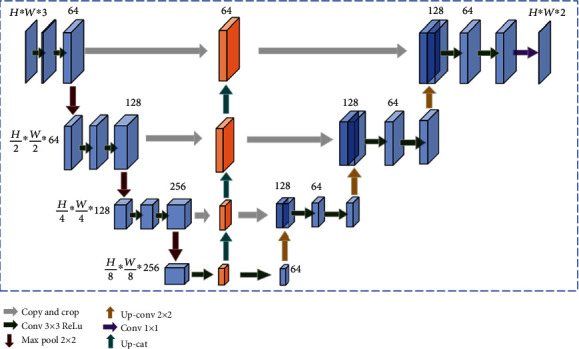
Detailed structure of A2 segmentation network.

In the second stage of the cascade framework, the U-shaped structure is used for segmentation in response to the specificity of medical image information features that are difficult to extract. Its jump succession strengthens the feature conduction of the whole network and alleviates the gradient disappearance problem while enhancing feature reuse and improving the learning ability of the network. In addition, we embedded an FPN module in this stage for accurate kidney tumor segmentation according to the local region in A1. The detailed network architecture of A2 is shown in Figure 5.

The encoding–decoding structure constitutes the main structure of the segmentation network. Due to the low resolution of the tumor in kidney images, the unit voxel contains information far beyond the natural image detail information; the voxel capacity is at least 16 bits or more. Therefore, we reduced it by one layer on top of the U-Net to prevent model overfitting. We performed three downsampling and three linear interpolation upsampling operations. The image input size was *H* × *W* × 3, and the output size was *H* × *W* × 2, where *H* and *W* represent the image length and width, respectively. The encoder network, shown on the left side in Figure 5, was used to perform a series of downsampling operations through convolution and max pooling, and in each subsequent downsampling operation, the number of feature channels was doubled. Two 3 × 3 convolutions were performed, each with a ReLU activation function and a 2 × 2 max pooling for downsampling in steps of 2. The number of feature maps was multiplied by 2 after each downsampling; thus, there was a change in the size of the feature map. The decoding section is shown on the right side in Figure 5; each step included the feature map for upsampling, followed by a 2 × 2 convolution that halved the number of feature channels and two 3 × 3 convolutions, again each followed by a ReLU activation function. In the final layer, a 1 × 1 convolution was used to map each 64-component feature vector to the desired number of classes.

**Figure 6:**
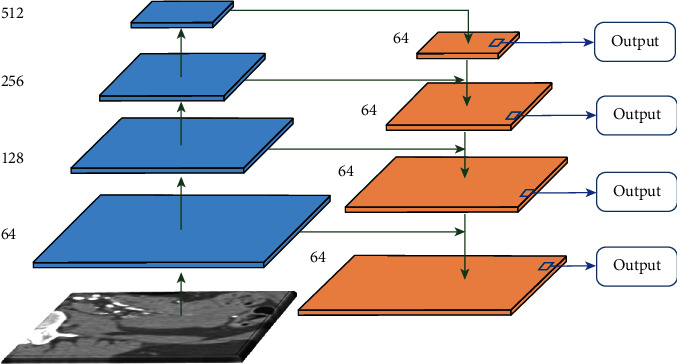
Feature pyramid network structure diagram.

Because the varying sizes of kidney tumors increase the difficulty of segmentation, while existing algorithms use expanded convolution (ASPP) or pyramidal convergence (PSPNet) to increase the accuracy of segmentation, they suffer from grid effects and loss of pixel-level location information, respectively, which are not conducive to the local consistency of feature mapping. Moreover, the pyramid pooling module employed in PSPNet loses pixel localization in pooling operations at different scales. To make full use of features and enhance feature extraction and propagation, in this study, the FPN module was incorporated because it can perform feature extraction for each scale of the image and is capable of producing multiscale feature representations, and all levels of feature maps have strong semantic information, including some high-resolution feature maps, which ultimately improve the segmentation efficiency of tumor regions. The FPN module details are shown in Figure 6.

The FPN structure consists of a bottom-up path, a top-down path, and a lateral connection. The backbone of the bottom-up path is the forward feedback computation of ConvNet, which computes a feature layer structure consisting of multiscale feature maps in steps of 2. In this study, a pyramid level was also defined for each stage, and the output of the last layer of each stage was selected as the reference feature map set. The top-down path obtains higher-resolution features by upsampling spatially coarser but semantically more powerful feature maps from higher pyramid levels. These features are then augmented by lateral connections from the bottom-up path. Each lateral connection merges feature maps of the same spatial size from the bottom-up path and the top-down path. In this study, for coarser-resolution feature maps, the spatial resolution was upsampled by a factor of 2. Then, the upsampled maps were merged with the corresponding bottom-up maps by performing pixel-by-pixel addition. The process was repeated until the finest-resolution map was generated.

### 3.3. Loss Function

The cross-entropy loss function is extensively employed to supervise model signals in medical image segmentation [24]. The cross-entropy loss function avoids the problem that the derivative form of the Sigmoid-type function is prone to saturation. In addition, the cross-entropy loss function can avoid gradient dispersion when performing gradient descent calculations, which leads to a decrease in the learning rate. Therefore, in this study, the cross-entropy loss function was used to calculate the segmentation loss:

(1) $$\begin{array}{c} L_{\text{CE}}\left(p,q\right)=-\overset{N}{\underset{\text{i}=1}{\sum }}p\left(x_{i}\right)\log\left(q\left(x_{i}\right)\right), \end{array}$$

where *N* denotes the number of categories, *p*(*x*_*i*_) denotes the true distribution of the sample, and *q*(*x*_*i*_) represents the distribution predicted by the model and the probability that the sample belongs to category *N*. *p*(*x*_*i*_) = 1 when the predicted category is the same as the category of the sample; otherwise, *p*(*x*_*i*_) = 0.

## 4. Experimental Details and Results

To illustrate the effectiveness and generalization ability of the proposed FYU-Net model, it was compared with mainstream models on a competition public dataset. The experimental results were analyzed from multiple perspectives. The proposed model was implemented using the PyTorch framework, and the running environment was a single NVIDIA GeForce GTX 3080 GPU with 10 GB of video memory. The epoch was set as 100 so that all models could reach convergence. The learning rate was set as 0.01, the weight decay rate was 0.0005, and the small batch stochastic gradient descent method with a momentum value of 0.9 was employed as the optimization method. In the first stage of the A1 network, we inputted the original image size in the “jpg” format. In the second phase of the experiment, the results obtained in the A1 model were cropped to include the slices of kidney tumors and then inputted into the A2 network.

### 4.1. Dataset

We used the dataset from the Kidney PArsing Challenge 2022 [25–28]. The dataset includes unenhanced CT images of the kidney from 70 patients. The tumors are multisubtype lesions, with five subtumor types in the dataset with variable distribution, resulting in a more challenging situation. First, we set the appropriate window width and bit value for the medical image data. Next, we converted the image format from “.nii.gz” to “jpg” and used the “jpg” image as the input of the model. Finally, the data were randomly divided into training and test sets in the ratio of 8 : 2.

### 4.2. Evaluation Indicators and Parameter Settings

To accurately evaluate the segmentation performance with reference to the current mainstream evaluation standards, we adopted four metrics, namely, Dice coefficient, Jaccard coefficient, Precision, and Recall:

(2) $$\begin{array}{c} \text{Dice}=\frac{2*\left\|V_{s}\cap V_{g}\right\|}{\left\|V_{s}+V_{g}\right\|}, \end{array}$$

(3) $$\begin{array}{c} \text{Jaccard}=\frac{\left\|V_{s}\cap V_{g}\right\|}{\left\|V_{s}\cup V_{g}\right\|}, \end{array}$$

(4) $$\begin{array}{c} \text{Precision}=\frac{\left\|V_{s}\cap V_{g}\right\|}{\left\|V_{s}\right\|}, \end{array}$$

(5) $$\begin{array}{c} \text{Recall}=\frac{\left\|V_{s}\cap V_{g}\right\|}{\left\|V_{g}\right\|}, \end{array}$$

where *V*_*s*_ denotes the set of pixels that automatically segment the kidney tumor and *V*_*g*_ denotes the true set of pixels in the kidney tumor region.

### 4.3. Analysis of Results

To verify the effectiveness of the proposed model, we compared it with the mainstream segmentation models, namely, APCNet, HRNet [29], FCN [30], and U-Net. The training parameters of these models were set according to their optimal parameters in the corresponding literature, and the recognition results were reacquired based on the source code provided by the authors. Qualitative evaluation and quantitative comparison with mainstream models demonstrated the effectiveness of the proposed FYU-Net model. Furthermore, model convergence analysis demonstrated the stability of the proposed model.

**Figure 7:**
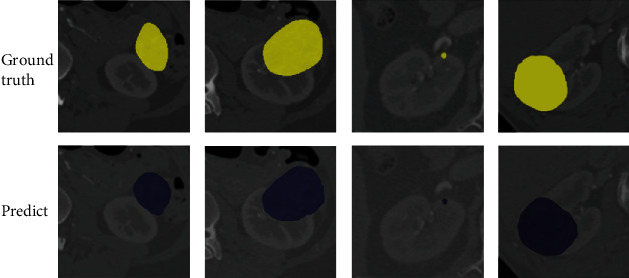
FYU-Net model segmentation results.

As shown in Figure 7, the proposed FYU-Net model exhibited an extremely high visual advantage, demonstrating that it can be extremely helpful for kidney tumor resection surgery. In Figure 7, the first row shows the true value of the kidney tumor, and the second row shows the FYU-Net segmentation result map. It can be observed that the proposed model achieved good tumor segmentation results visually.

**Figure 8:**
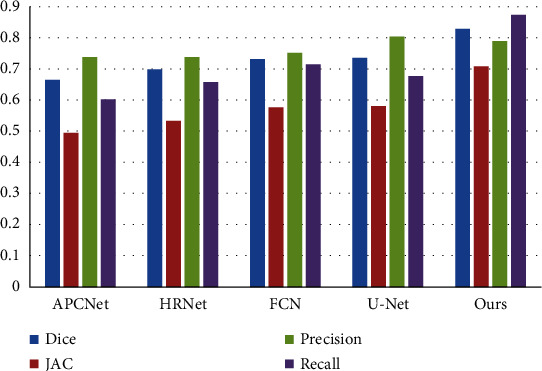
Visualization of model comparison results.

The experimental results of the proposed model and mainstream models on the kidney dataset are presented in Table 1. The proposed model achieved the maximum Dice coefficient, Jaccard coefficient, and recall values, which were 82.85%, 70.73%, and 87.38%, respectively, and its recognition performance was considerably better than that of other models. Precision represents the probability of tumor detection in the prediction result; however, a high precision value alone does not indicate that the model's segmentation effect is good. In the proposed model, the FCN was used to replace the fully connected layer with a convolutional layer, and deconvolution was used for upsampling. In addition, a jump-connected structure was used to combine coarse information with fine information for segmentation. Thus, the tumor segmentation effect of the proposed model was better than that of APCNet and HRNet. The Dice coefficient and Jaccard coefficient values for the proposed model were 73.13% and 57.65%, respectively. In contrast, U-Net differs from FCN; it extends the FCN network structure, and its upsampling still has a large number of channels, which allows the network to propagate the contextual information to higher resolution, and the upsampling part fuses the output of the feature extraction part, which fuses the multiscale features. Thus, U-Net achieves better segmentation results than FCN, with Dice coefficient, Jaccard coefficient, and precision values of 73.50%, 58.10%, and 80.39%, respectively.  Figure 8 illustrates the visualization comparison results.

### 4.4. Ablation Experiments

To better validate the effectiveness of the proposed FYU-Net model, we conducted numerous ablation experiments. As presented in Table 2, we compared the segmentation results of FYU-Net without adding various strategies; SHU-Net denotes shallow U-Net, and LN denotes localization network. Our improved strategies resulted in considerable performance improvement. The segmentation Dice and Jaccard coefficient values of the original U-Net on the renal tumor data were 73.50% and 58.10%, respectively. The Dice and Jaccard coefficient values increased to 75.13% and 60.17%, respectively, when we used only the FPN strategy. When both the shallow U-Net structure and the FPN module were employed, the segmentation effect improved, and the Dice and Jaccard coefficient values increased to 76.49% and 61.92%, respectively. When we adopted the full strategy and used FYU-Net for segmentation of kidney tumor CT data, the segmentation effect improved considerably, resulting in an average Dice coefficient of 82.85% and a Jaccard coefficient of 70.73%, thus demonstrating the effectiveness of our improvement scheme.

### 4.5. Model Convergence Analysis

**Figure 9:**
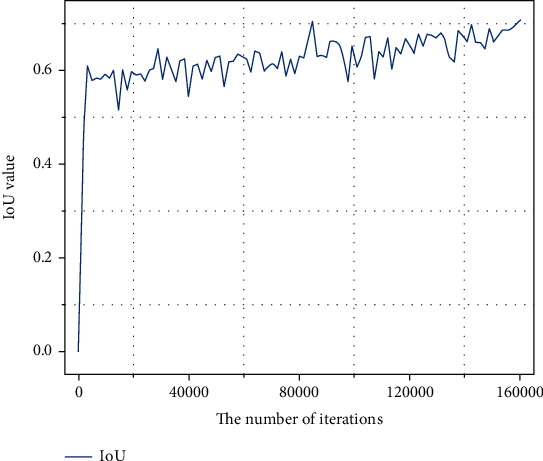
IoU convergence trend of FYU-Net model.

The number of training iterations of the model is one of the important factors affecting the segmentation structure of kidney tumors. As shown in Figure 9, the IoU of the proposed model started to increase abruptly from 0 to 0.6, reflecting the effectiveness of FYU-Net. As the number of iterations increased, the IoU curve exhibited a gradual growth trend and finally reached a convergence state, with the IoU value finally stabilizing at around 0.7. Due to the video memory, we set the batch_size as 2, thus causing the IoU curve to still have a small oscillation late in the iteration.

## 5. Discussion

In this study, we developed a cascaded segmentation network for medical images to realize tumor region segmentation in kidney CT data. Because tumors are irregularly shaped and relatively small in volume compared to the kidney and may appear at different locations in the kidney, we first used a target detection algorithm based on the YOLO-V5 m implementation to quickly localize tumor regions in the raw kidney CT data. Next, we applied it to localized regions to perform the final accurate segmentation of the tumor. Experimental results on the publicly available kidney CT dataset provided by Kidney PArsing Challenge 2022 demonstrated that the proposed model is more accurate and robust than existing methods. The Dice coefficient and Jaccard coefficient values for the proposed model were 82.85% and 70.73%, respectively. Furthermore, the FYU-Net model proposed in this paper demonstrated good time efficiency, indicating that the proposed cascaded segmentation network greatly improves the efficiency of medical image segmentation. Finally, ablation experiments demonstrated that the proposed network yields superior medical image segmentation performance.

## Figures and Tables

**Table 1:** Segmentation results of each model on kidney tumor data.

Model	Dice	Jaccard	Precision	Recall
APCNet	0.6627	0.4956	0.7374	0.6017
HRNet	0.6957	0.5334	0.7389	0.6573
FCN	0.7313	0.5765	0.7490	0.7145
U-Net	0.7350	0.5810	**0.8039**	0.6770
Ours	**0.8285**	**0.7073**	0.7877	**0.8738**

**Table 2:** Comparison results of ablation experiments.

SHU-Net	LN	FPN	Dice	Jaccard
—	—	—	0.7350	0.5810
—	—	√	0.7513	0.6017
√	—	√	0.7649	0.6192
√	√	√	**0.8285**	**0.7073**

## Data Availability

The data used in this study is the public dataset provided by Kidney PArsing Challenge 2022, which can be obtained by registering for the contest or by accessing the data through the authors' Github website (https://github.com/L9uckin/kidney).

## Additional Points

*Code Availability.* The code used for segmentation of kidney tumors from CT data are available from the authors' Github website (https://github.com/L9uckin/kidney). The codes used for the other computational analyses are available upon request to the authors.
